# Assessment of Citations of the Retracted Article by Wakefield et al With Fraudulent Claims of an Association Between Vaccination and Autism

**DOI:** 10.1001/jamanetworkopen.2019.15552

**Published:** 2019-11-15

**Authors:** Elizabeth M. Suelzer, Jennifer Deal, Karen L. Hanus, Barbara Ruggeri, Rita Sieracki, Elizabeth Witkowski

**Affiliations:** 1 Medical College of Wisconsin Libraries, Medical College of Wisconsin, Milwaukee; 2 Ziebert Medical Library, Advocate Aurora West Allis Medical Center, West Allis, Wisconsin; 3 Todd Wehr Library, Carroll University, Waukesha, Wisconsin

## Abstract

**Importance:**

The number of citations can be used to show the influence of an article or to measure the validity of a research study. The article by Wakefield et al that fraudulently reported an association between vaccination and autism continues to accumulate citations even after it was retracted.

**Objectives:**

To examine the characteristics of citations from scholarly literature that reference the 1998 article by Wakefield et al and to investigate whether authors are accurately citing retracted references.

**Design, Setting, and Participants:**

In this cross-sectional bibliographic analysis of the scholarly publications that cited a 1998 article by Wakefield et al, cited references were collected from a Web of Science Core Collection search performed on March 11, 2019. A total of 1211 articles were identified, with 58 citing works excluded because they were non–English-language publications or the citation to the study by Wakefield et al could not be located by reviewers. Citing works consisted of books, research articles, letters, editorials, news items, and other scholarly literature. Citations to the article by Wakefield et al were identified and analyzed by 2 reviewers in a blinded screening. Reviewers assigned a characteristic to each citation and indicated whether the retraction was documented.

**Main Outcomes and Measures:**

The characteristics of citations to the article by Wakefield et al, were categorized as negative, affirmative, or contrastive; if not, persuasive; and if not, assumptive, perfunctory, methodologic, or conceptual. Whether the partial retraction or notice of retraction was included in the citing work was also documented.

**Results:**

Among the 1153 citing works included in this analysis, the most common citation characteristics were negative (838 [72.7%]) followed by perfunctory (106 [9.2%]) and affirmative (94 [8.2%]). A total of 123 of 322 citing works (38.2%) published between 2005 and 2010 documented the partial retraction. After the notice of retraction was published in 2010, the percentage of citing works that documented the partial retraction and/or notice of retraction between 2011 and 2018 increased to 360 of 502 (71.7%).

**Conclusions and Relevance:**

Since the article by Wakefield et al was initially published, authors have mostly negated the findings of the study. A significant number of authors did not document retractions of the article by Wakefield et al. The findings suggest that improvements are needed from publishers, bibliographic databases, and citation management software to ensure that retracted articles are accurately documented.

## Key Points

**Question:**

What are the characteristics of citations of the retracted 1998 article by Wakefield et al that purported to show an association between the measles-mumps-rubella vaccine and autism?

**Findings:**

In this cross-sectional bibliographic analysis of 1153 works citing the article by Wakefield et al, citation characteristics were mostly negative, but since the notice of retraction was issued in 2010, many of the citing works published afterward did not indicate that the article was retracted.

**Meaning:**

The findings suggest that improvements are needed from publishers, bibliographic databases, and citation management software to ensure that retracted articles are accurately documented.

## Teaser

This cross-sectional study analyzes the characteristics of citations from scholarly literature that reference the 1998 article by Wakefield et al and investigates whether authors are accurately citing retracted references.

## Introduction

In an era of information overload, it can be challenging to find influential articles in any given field, and one method of identifying such influential articles is to look at citation counts. High citation counts are often equated with articles with high impact factors,^1,2,3^ but “impact is not the same as importance or significance.”^4^^(p 290)^ The raw number does not show the intent of an author in deciding to cite another’s work, and there can be a variety of motivations behind the intent.^5,6^ For instance, citing a work in a negative manner can be used by researchers to self-correct flawed research and aid in scientific debate.^7^ Value would be added to citation count numbers if more information was provided concerning the context of the citations.^5,6^ However, obtaining information about citation characteristics is laborious, and using natural language processing is a relatively new and emerging field.^7,8^

Retracted articles continue to be cited frequently without listing the retraction,^9,10^ perhaps because there are no clear guidelines on whom is responsible for ensuring that retracted articles are properly cited as being retracted.^11^ The Committee on Publication Ethics (COPE) states, “Retracted articles should be clearly identified as such in all electronic sources,”^12^^(p 202)^ but the committee does not offer guidelines on how and when authors should document that an article has been retracted. The International Committee of Medical Journal Editors (ICMJE) notes that not all journals check the accuracy of references in submitted publications.^13^ The omission of a work’s retracted status in the citation can lead to the perception that the cited work is valid,^14^ requiring scholars to spend time, effort, and money to correct the misperceptions of the problematic work.^15^

The purpose of this cross-sectional study was to examine how authors of scholarly literature cited the 1998 article by Wakefield et al^16^ that purported to show an association between the measles-mumps-rubella (MMR) vaccine and autism. The article was retracted in 2 stages: in 2004, 10 of the original 12 authors issued a partial retraction of the interpretation that there is a causal link between the MMR vaccine and autism (1 of the original authors was unable to be contacted and Wakefield was not an author included in this partial retraction),^17^ and in 2010, editors of *The Lancet* published a notice of retraction of the article because of false claims made in the original article.^18^ Despite the partial retraction and notice of retraction, the 1998 article by Wakefield et al^16^ continues to accumulate a significant amount of citations. In 2014, of the 58 million references in Web of Science, only 14 499 works (0.026%) have been cited more than 1000 times.^19^ The 1998 article by Wakefield et al^16^ had accumulated more than 1211 citations by March 2019, and the number continues to increase. According to the Web of Science Core Collection, in April 2019, the article by Wakefield et al^16^ was the ninth most-cited reference indexed with the topic of autism of more than 57 600 references and the second most-cited reference indexed with the topic of measles vaccines of more than 900 references. The present study was not the first citation analysis of the article by Wakefield et al.^16^ For instance, Chen and Leydesdorff^8,20^ used citation data from Web of Science to look for patterns in how journals and research fields cited the retracted article by Wakefield et al.^16^ However, these patterns do not examine the context of the citations. The present study is novel because it is the first time, to our knowledge, that each citation to the article by Wakefield et al^16^ was analyzed to see how the author cited the article, specifically assessing whether the author affirmed or negated the study by Wakefield et al.^16^ Reviewers examined the citing works to evaluate whether the retracted status of the article by Wakefield et al^16^ was identified in the citation or reference list.

This study replicated the methods used by Leung et al^21^ in their article, *1980 Letter on the Risk of Opioid Addiction*. The conclusion of Leung et al^21^^(pp 2194-2195)^ highlights “the potential consequences of inaccurate citation and underscores the need for diligence when citing previously published studies.” Reviewers planned to use the methods of Leung et al^21^ to examine another influential article of questionable quality. Terms such as *reference* and *citation* are sometimes used interchangeably; thus, a glossary of terms is given in Table 1.

**Table 1.** Glossary of Terms Used in This Article

Term	Definition
Reference	Original work that is documented in another work as a line item in a bibliography or as a footnote
Citation	Documentation of the reference; in other words, the in-text mention of a work
Cite	Act of referencing another’s work
Cited reference	Line item in a bibliography that gives credit to the original work
Cited work	Specific item being cited
Citing work	Publication that contains a citation
Notice of retraction	Documentation from the editor stating that a publication has been retracted
Partial retraction	Documentation from an editor or author stating that a portion of the published work has been retracted
Retracted article	Article that has been formally withdrawn by the publisher because of fraud, error, misconduct, or redundancy^12^
Self-citation	Act of an author citing his or her previous work as a reference in subsequent works

## Methods

In this cross-sectional study, we (E.M.S., J.D., K.L.H., B.R., R.S., and E.W.) conducted a cited reference search on March 11, 2019, in the Web of Science Core Collection to identify scholarly literature that cited the 1998 article by Wakefield et al.^16^ The reviewers’ Web of Science Core Collection subscription includes access to Science Citation Index Expanded, Social Science Citation Index, Arts & Humanities Citation Index, and Book Citation Index, allowing the reviewers to find cited references that span all scholarly disciplines, including clinical medicine, social sciences, immunology, and neurology and behavior. Web of Science indexes research articles, letters, editorials, news items, proceedings literature, books, and other scholarly literature from sources that demonstrate high levels of editorial rigor and meet a well-defined set of criteria,^22^ thus allowing a good representation of the scholarly community’s reaction to the 1998 article by Wakefield et al.^16^ The Cited Reference Search feature in Web of Science allows for a comprehensive search of bibliographies and reference lists of all items that are indexed in Web of Science. This study followed the Strengthening the Reporting of Observational Studies in Epidemiology (STROBE) reporting guideline.^23^ The Medical College of Wisconsin Institutional Review Board Office reviewed this project and determined that this study did not qualify as human subjects research and was therefore not subject to institutional review board review.

We identified 1211 citations to the 1998 article by Wakefield et al^16^ as of March 11, 2019. Fifty-eight articles were excluded from the citation analysis because the works were not written in English or the citation to the article by Wakefield et al^16^ could not be located by the reviewers. A total of 1153 citing works were included in the citation analysis. Bibliographic information and the full-text copy of each citing work was uploaded into Covidence systematic review software (Veritas Health Innovation). Covidence is a web-based software platform that is used to manage the screening process and extract data for systematic reviews; it allows blinded screening and custom tagging of records.

Citing works were reviewed to determine the characteristic of the citation using an established taxonomy^5,21^ (Table 2). Each citing work underwent a blinded screening by 2 of us (E.M.S., J.D., K.L.H., B.R., R.S., and/or E.W.) who located the citations within the text of the work and independently assigned them a characteristic. Disagreement about the characteristics were brought before the group (E.M.S., J.D., K.L.H., B.R., R.S., and E.W.) for consensus. If citations fit into more than 1 category or the article by Wakefield et al^16^ was cited more than once, a stepwise approach was used to assign the category.^21^ In the stepwise approach, citations were first screened to assess whether they fit in the categories of negative, affirmative, or contrastive; if not, they were screened for the category of persuasive; if not, citations were screened for the categories of assumptive, perfunctory, methodologic, or conceptual. If a citation could not be located, the citing work was excluded from the review. For multiple citation occurrences, the citation was categorized as contrastive if any of the citations had this characteristic. Barring this, a citation was categorized as overall negative or affirmative if any of the occurrences had these characteristics. Finally, if citations did not meet any of these criteria, the citing works were brought before the group for consensus. Every citing work in the sample was assigned one characteristic.

**Table 2.** Citation Characteristics^a^

Characteristic	Definition
Affirmative	Citing work confirms, is supported by, depends on, agrees with, or is strongly influenced by cited work
Assumptive	Citing work refers to assumed knowledge that is general or specific background or an historical account or acknowledges cited work pioneers
Conceptual	Citing work uses definitions, concepts, or theories of cited work
Contrastive	Citing work contrasts between the current work and cited work or other works with each other or is an alternative to cited work
Methodologic	Citing work uses materials, equipment, practical techniques, tools, analysis methods, procedures, or design of cited work
Negative	Citing work disputes, corrects or questions, or negatively evaluates cited work
Perfunctory	Citing work makes a perfunctory reference, is cited without additional comment, makes a redundant reference to cited work, or is not apparently strictly relevant to the author’s immediate concerns
Persuasive	Cited work is cited in a ceremonial fashion or is authored by a recognized authority in the field

^a^ Adapted from definitions by Bornmann and Daniel.^5^

Not only were the citing works screened to determine the characteristic of each citation, they also were examined to determine whether the retracted status of the article by Wakefield et al^16^ was identified in the citation or in the reference list. A citing work was labeled as *retraction referenced* if the author specifically used the word *retracted*, *retract*, or *retraction*.

### Statistical Analysis

Bibliographic information, characteristics, and retraction information were exported from Covidence as a CSV file, and Excel (Microsoft Corp) was used to analyze the data. A descriptive analysis of the data was performed with a focus on frequencies and percentages. The sample consisted of all the included citing works retrieved from the Web of Science search apart from the 58 excluded articles.

## Results

### Characteristics

**Figure 1.**
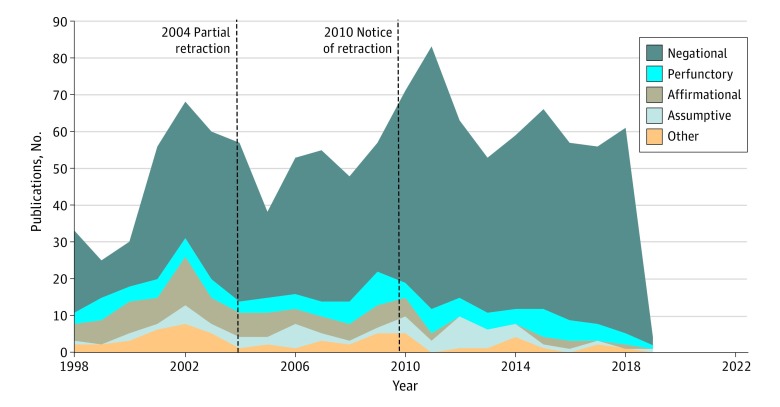
Characteristics of References to the Article by Wakefield et al by Year of Publication Other characteristic categories include conceptual, contrastive, methodologic, and persuasive and include a total of 55 publications. A partial retraction to the article by Wakefield et al^16^ was published in 2004,^17^ and another retraction was published in 2010.^18^

Of the 1153 citing works, 838 (72.7%) were negative, 106 (9.2%) perfunctory, 94 (8.2%) affirmative, and 60 (5.2%) assumptive. The other characteristics (conceptual, contrastive, methodologic, and persuasive) had a combined total of 55 (4.8%). Since the article by Wakefield et al^16^ was initially published, authors have mostly cited the article in a negative manner (Figure 1). Authors who affirmed the article by Wakefield et al^16^ in their citations comprised 94 of the 1153 total citing works (8.2%). Of the affirmative citations, 49 of 94 (52.1%) were published between 1998 and 2003, before the partial retraction of the article by Wakefield et al.^16^ Wakefield was an author of 15 of 94 articles with affirmative citations. Of these 15 affirmative self-citing works, 10 include at least 1 coauthor of the original article. The only other self-citing work by the original authors of the article by Wakefield et al^16^ is the 2004 partial retraction by Murch et al,^17^ which was characterized as negative.

### Retractions

**Figure 2.**
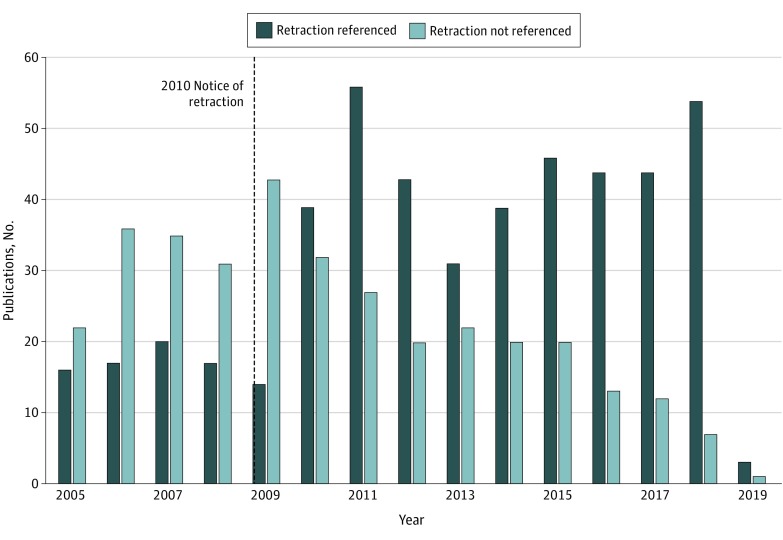
Number of Articles That Referenced the 1998 Article by Wakefield et al by Year After the Partial Retraction and Notice of Retraction Were Published Retractions to the article by Wakefield et al^16^ were published in 2004 and 2010.^17,18^

From the date of the partial retraction on March 4, 2004, to March 11, 2019, a total of 881 works were published that cited the article by Wakefield et al.^16^ These citing works were screened to determine whether they documented the partial and/or notice of retraction to the article by Wakefield et al,^16^ and in this period, 493 of 881 (56.0%) of the citing works documented either retraction. Of 57 citing works published in 2004, a total of 10 (17.5%) documented the partial retraction in the citations or the reference lists. However, the reviewers began the retraction analysis (Figure 2) in 2005 to compensate for works that were submitted for publication before the partial retraction was published and to ensure that the retraction information was indexed in bibliographic databases, making it more likely to be discovered. Between 2005 and 2010, a total of 123 of 322 (38.2%) citing works documented the partial retraction. In 2010, editors of *The Lancet* issued a notice of retraction^18^ to the article by Wakefield et al.^16^ Of the 71 citing works published in 2010, a total of 21 (29.6%) documented the 2010 notice of retraction or retracted status of the article. The number of authors who documented the partial retraction or the notice of retraction between 2011 and 2018 increased to 360 of 502 (71.7%). Since 2013, the percentage of authors who have documented either retraction continually improved, and in 2018, a total of 54 of 61 citing works (88.5%) documented either retraction.

We found that authors documented the retraction in different ways. Of the 493 citing works that documented either retraction, 211 (42.8%) documented the retraction in both the citation and the reference list; 189 (38.3%) documented the retraction in their citations, but the reference to the study by Wakefield et al^16^ in the reference list did not include a variation of the word *retract* or a reference of the notice of retraction or of the partial retraction; and 93 (18.9%) documented either retraction in the reference list or included the word *retract* in the reference to the article by Wakefield et al^16^ but not in the citation.

## Discussion

The first aim of this study was to assign a characteristic to each citing work of the article by Wakefield et al^16^ to determine how authors cited the information. Most authors cited the article by Wakefield et al^16^ in a negative manner. Even before the article was formally retracted, authors were asserting that there were problems with the study, including the small sample size^24,25,26^ and the lack of epidemiologic evidence to support the increased risk of autism after receiving the MMR vaccine.^27,28,29^ Later, authors reported that the conclusions of the article by Wakefield et al^16^ were not reproducible.^30,31,32^ Soon after publication of the study, scholars may have recognized that it was flawed. Thus, although this was a highly influential article, it is an example of how high citation count may not equate with a high-quality work.

Articles that affirmed the article by Wakefield et al^16^ in their citations comprised only 8% of the total cited references. There were common themes among these citing works. Some authors stated that the article confirmed parents’ observations of their autistic children,^33,34,35^ others affirmed the gut-brain connection to autism,^36,37,38^ and others cited the article by Wakefield et al^16^ in their own article’s introduction or discussion section as a reason to support their research studies on gut diseases.^39,40,41^

The second aim of this study was to record whether the retractions were accurately documented. The ICMJE recommendations for manuscript preparations suggest that authors are responsible for ensuring that reference lists are accurate, that authors use PubMed as an authoritative source for information about retractions, and that authors should note the retracted status of the article in the citation when citing a retracted article.^13^ Despite these recommendations, 142 of the 502 citing works (28.3%) published after 2011 did not document either retraction or note the retracted status in the citation.

There are challenges in identifying and documenting retracted literature. First, bibliographic databases have differing policies on indexing retractions. According to Web of Science (Product Support, Clarivate Analytics, written communication, September 25, 2019), if a work is specifically identified as having been retracted, the title of the original item is updated to include “Retracted Article” along with the published notice of the retraction’s citation information. The Document Type “Retracted Publication” is added to the record for the original item. The notice of retraction is also indexed using the same title and authors as the original item so that any title or author search that retrieves the original will also retrieve the notice of retraction. The title of the notice of retraction is also updated to include “(Retraction of)” along with the retracted item’s citation information. The Document Type “Retraction” will be added to the record of the notice of retraction.

Because Web of Science alters the original publication’s record by adding the words “retracted article” in the title of the original publication and by including “retraction of” in the record for the notice of retraction, the work’s retracted status is evident. Even after downloading into citation management software, the title of the retracted work includes “retracted article,” making it hard to overlook the retracted status.

In contrast to Web of Science, PubMed connects citations for original articles and citations for notices of retraction based on information supplied by the journal publisher within their database and includes a banner that indicates that the publication is retracted but does not change the title of the original publication. “The Publication Type ‘Retraction of Publication’ is assigned to the citation for the retraction notice, and the citation is linked to the citation for the retracted article. The Publication Type ‘Retracted Publication’ is assigned to the retracted article. Citations for retraction notices contain the phrase ‘Retraction of: [article title],’ and citations for retracted articles contain the phrase ‘Retraction in: [article title].’”^42^

Although it is clear that an article has been retracted when searching within PubMed, after downloading and/or depositing into citation management software, only the publication type identifier indicates that the work has been retracted. Many information users may overlook this indication.

Second, journals have differing policies on how they update articles that have been retracted. For example, *JAMA* adds a watermark with the word *retraction* in red letters to the article record on their website, and the PDF versions of retracted articles are marked with a header to alert the reader that the article has been retracted, per COPE recommendations.^12^ However, when the article information is downloaded into citation management software, the retracted status is not evident. *The Lancet*, on the other hand, updates the item record of the article to include the word *retracted* at the beginning of the title. When article information is downloaded into citation management software, the retraction information is labeled. Changing the article title makes it easier for bibliographic databases to capture the retraction information as well. We recommend that publishers add a label, such as “retracted,” to the title of articles that have been issued notices of retraction.

Third, not all citation styles provide guidelines for citing retractions. The *Publication Manual of the American Psychological Association*, sixth edition^43^ does not contain instructions for citing a retracted article. The *AMA Manual of Style* has a reference style for retractions, but citation management software, such as EndNote, RefWorks, and Zotero, do not automatically include the additional information required by the *AMA Manual of Style*.^44^ According to the AMA citation style,^44^ the citation to the 1998 article by Wakefield et al^16^ should appear as follows:

1. Wakefield AJ, Murch SH, Anthony A, et al. Ileal-lymphoid-nodular hyperplasia, non-specific colitis, and pervasive developmental disorder in children [retracted in: *Lancet*. 2010;375(9713):445]. *Lancet*. 1998;351(9103):637-641.

The text within the brackets was manually added to the reference by the authors. In this example, only the notice of retraction was documented. It is not clear from the *AMA Manual of Style* whether notice of partial retraction should also be included in the citation. Authors may not be aware that their citation management software is not properly citing a retracted article, and journal editors and article reviewers are likely relying on authors to ensure that their citations are correct.

Fourth, a retraction can occur after a bibliographic reference has been downloaded into citation management software; thus, an information user may not be aware of a subsequent retraction. Zotero^45^ now includes an enhancement that will identify retracted articles that have been downloaded into a Zotero library, but not all citation management software includes this feature. The retracted status of the article by Wakefield et al^16^ is well known in the scholarly community, but authors failed to cite it accurately. To ensure the integrity of scholarly articles and research, better care needs to be taken to ensure that retracted articles are properly cited.

### Limitations

This study has limitations. The reviewers relied solely on Web of Science to identify citing works of the 1998 article by Wakefield et al.^16^ In addition, assigning a single characteristic to a citing work was challenging and subjective.

## Conclusions

Although the 1998 article by Wakefield by al^16^ continues to accumulate citations, most works that cited it did so in a negative manner. The retracted status of the article by Wakefield article et al^16^ is well known in the scholarly community, but the findings suggest that authors failed to cite it accurately. To ensure the integrity of scholarly articles and research, we believe that better care needs to be taken to ensure that retracted articles are properly cited and that it is ultimately the authors’ responsibility to ensure that their citations are accurately documented. Improvements can be made to the indexing procedures of bibliographic databases, journal publisher procedures for updating retracted articles, and citation management software products to make it more apparent when articles have been retracted. In addition, stronger guidelines from the ICMJE, COPE, and citation styles on how to cite retracted articles appear to be needed. We also believe that authors should take additional steps to verify their citations by using bibliographic databases, such as PubMed and Retraction Watch, or enlisting the help of librarians and that journal editors should hold authors more accountable for checking their references.
